# Reward contingency reorganizes lateral orbitofrontal cortex pyramidal neuron activity from reward-aligned to action-aligned during associative learning

**DOI:** 10.3389/fncel.2026.1850197

**Published:** 2026-08-21

**Authors:** Zicheng Yang, Xiaolan Chen, Xiaohong Sun, Huimeng Lei

**Affiliations:** School of Basic Medical Sciences, Beijing Key Laboratory of Neural Regeneration and Repair, Key Laboratory for Neurodegenerative Diseases of the Ministry of Education, Laboratory for Clinical Medicine, Capital Medical University, Beijing, China

**Keywords:** anticipatory licking, fiber photometry, instrumental conditioning, lateral orbitofrontal cortex, Pavlovian conditioning, pyramidal neuron, reward contingency

## Abstract

**Background:**

Animals must learn not only which cues predict reward, but also when to act to obtain it. Although the orbitofrontal cortex (OFC) encodes reward-related information, whether reward contingency is sufficient to reorganize the temporal alignment of OFC activity remains unclear.

**Methods:**

We recorded calcium signals from lateral OFC pyramidal neurons (lOFC-PNs) in wild-type (WT) and *Sapap3* knockout (KO) mice using fiber photometry, during sequential learning of Pavlovian and instrumental contingencies in a single-odor reward association task. We further used a cue-free lick test and linear mixed-effects modeling to dissociate the contribution of reward contingency from that of licking behavior.

**Results:**

Licking behavior and lOFC-PN activity were comparable between WT and KO mice, supporting pooled analysis. Under Pavlovian contingencies, licking and lOFC-PN activity were concentrated around reward delivery. Under instrumental contingencies, both shifted to the pre-reward delay period, and the timing of lOFC-PN activity became progressively coupled to anticipatory licking as learning advanced. A cue-free lick test and linear mixed-effects modeling confirmed that these contingency-related differences reflected reward contingency rather than licking *per se*.

**Conclusion:**

These findings indicate that reward contingency dynamically reorganizes the temporal alignment of lOFC-PN activity and its coupling with behavior, shifting from reward-aligned under Pavlovian learning to action-aligned under instrumental learning. This contingency-dependent temporal reorganization may support flexible adaptation of neural processing to changing reward contingencies during associative learning.

## Introduction

Survival in complex environments requires animals to associate sensory cues with rewards and to flexibly adjust behavioral strategies according to the causal relationship between actions and outcomes (Balleine and O'Doherty, 2010; Sutton and Barto, 1998). Beyond learning which cues predict reward, animals must also learn when to act to obtain it. Two fundamental forms of associative learning support this capacity. In Pavlovian conditioning, reward is delivered independently of behavior, whereas in instrumental conditioning, reward delivery depends on the animal’s actions (Rescorla and Solomon, 1967; Balleine and Dickinson, 1998; Balleine, 2011). These two forms differ in their underlying action-outcome contingencies and may impose distinct temporal and computational demands on neural circuits organizing reward-related behavior (Lee and Keramati, 2017; Simmler and Ozawa, 2019; Jenni et al., 2023; Piccin et al., 2024; Chandrasekaran et al., 2024).

The lateral orbitofrontal cortex (lOFC) has been identified as a central node in reward-based associative learning (Schoenbaum et al., 2009; Wallis, 2007; Ogg et al., 2025), with prior work emphasizing its role in encoding expected outcomes, reward value, and task states (Rudebeck and Rich, 2018; Liu et al., 2025; Howard et al., 2020). lOFC pyramidal neurons (lOFC-PNs) exhibit prominent activity around reward delivery (Stalnaker et al., 2015; Namboodiri et al., 2019; Riceberg and Shapiro, 2017; Izquierdo, 2017; van Wingerden et al., 2010; Romero-Sosa et al., 2025), and disruption of lOFC function impairs the ability to update learned associations when contingencies change, as evidenced by reversal learning deficits across species (Hamilton and Brigman, 2015; Rudebeck and Murray, 2014; Yang et al., 2021). Notably, this lOFC-dependent flexibility is selectively impaired in *Sapap3* knockout (KO) mice, a well-established model of obsessive-compulsive-related circuit dysfunction (Yang et al., 2021), although their performance under simpler associative learning paradigms remains less well characterized. However, whether this temporal organization of lOFC-PN activity is stable across different reward contingencies, or reorganizes systematically when the action-outcome relationship changes, remains unknown.

Prior studies that examined lOFC function across Pavlovian and instrumental tasks employed paradigms involving multiple sensory cues, distinct reward types, or graded outcome values, allowing them to address rich questions about outcome value, stimulus specificity, and behavioral flexibility (Ostlund and Balleine, 2007; Cazares et al., 2022; Li et al., 2026). However, whether reward contingency alone, independent of stimulus identity or reward value, is sufficient to reorganize the temporal dynamics of lOFC-PN activity therefore remains an open question.

Here, we investigated how Pavlovian and instrumental reward contingencies shape the temporal organization of lOFC-PN activity during associative learning. We (i) developed a single-odor reward association task to compare licking behavior under two reward contingencies, while recording lOFC-PN activity using fiber photometry; (ii) compared licking behavior and lOFC-PN activity between WT and *Sapap3* KO mice to test whether lOFC-PN function during this single-odor associative learning paradigm is preserved in this model; (iii) used a cue-free lick test and linear mixed-effects modeling to dissociate the contribution of reward contingency from that of licking *per se*; (iv) examined whether the timing of lOFC-PN activity became coupled to anticipatory licking with learning under each contingency; and (v) identified in which task epochs performance-related lOFC-PN activity was concentrated under Pavlovian and instrumental contingencies. Together, this study addresses the behavioral and neural dynamics underlying distinct reward contingencies, and the contingency-dependent coupling between behavior and neural activity, advancing our understanding of how the lOFC dynamically supports reward-guided learning.

## Result

### Result 1. Licking behavior and lOFC-PN activity are comparable between WT and *Sapap3* KO mice in an odor-reward association task

**Figure 1:**
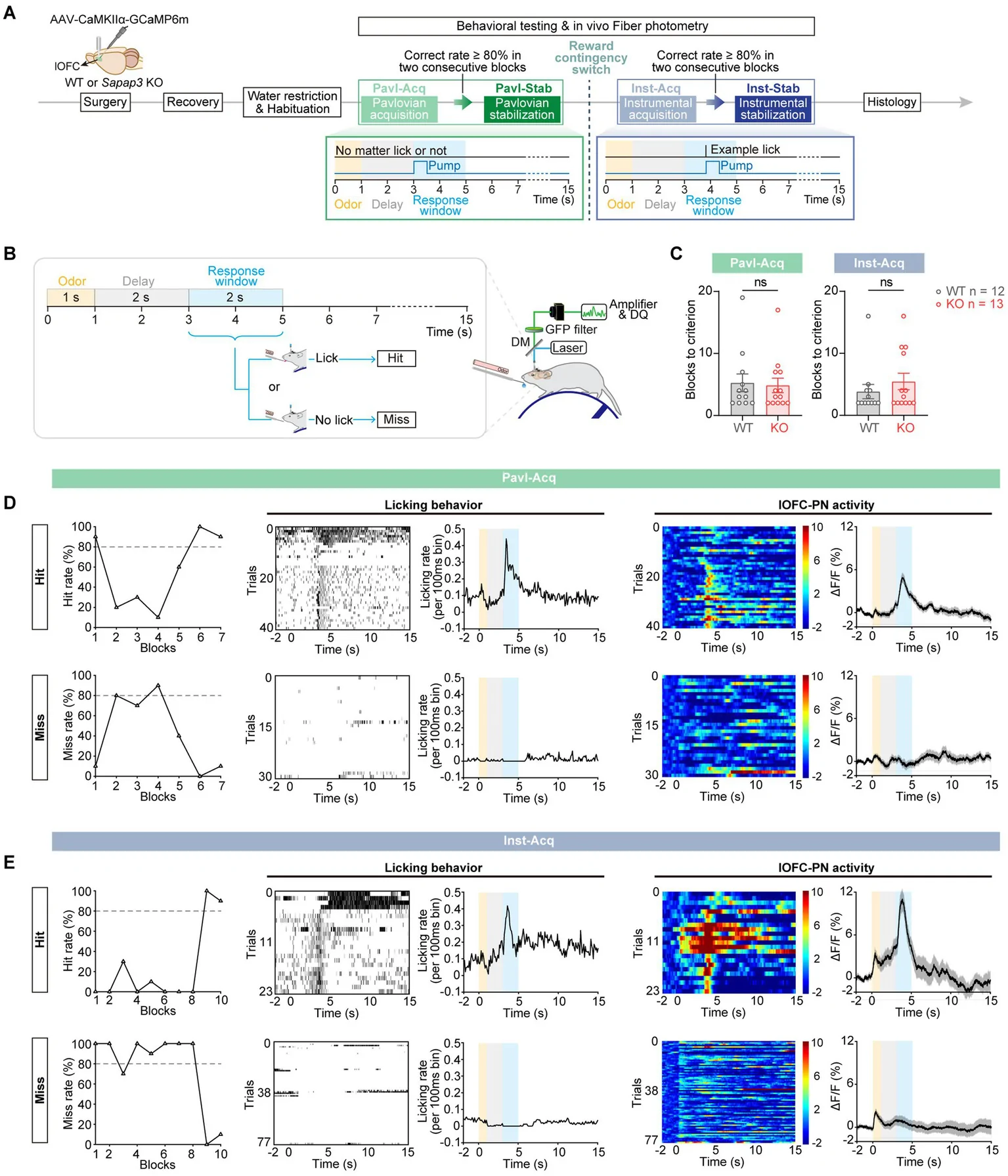
Odor-reward association task to dissect reward contingency from stimulus identity and reward value. **(A)** Experimental timeline. **(B)** Trial structure (left) and fiber photometry setup (right). **(C)** Number of blocks required to reach the behavioral criterion in the Pavl-Acq (left) and Inst-Acq (right) stages for WT (*n* = 12) and *Sapap3* KO (*n* = 13) mice. Each circle represents one mouse. Data are mean ± SEM. Mann–Whitney test. **(D,E)** Representative single-mouse data from the Pavl-Acq **(D)** and Inst-Acq **(E)** stages. From left to right: Block-wise hit (top) and miss (bottom) rates across the stage; lick rasters with average lick rate traces (per 100 ms bin); heatmaps of lOFC-PN calcium signals (ΔF/F) with average traces, shown separately for hit (top) and miss (bottom) trials. Shaded regions along the time axis indicate task epochs (odor, 0–1 s, yellow; delay, 1–3 s, gray; response window, 3–5 s, blue). Shaded areas around traces represent SEM.

To investigate how reward contingency reorganizes lOFC pyramidal neuron (hereafter lOFC-PN) activity during associative learning, we developed an odor-reward association task in which a single odor cue predicted reward delivery under either Pavlovian or instrumental contingencies (Figure 1A), thereby isolating reward contingency from stimulus identity and reward value as task variables.

To monitor lOFC-PN activity during behavior, we injected AAV-CaMKIIα-GCaMP6m into the lOFC and implanted an optical fiber over the injection site. After 3 weeks of viral expression and recovery, WT and *Sapap3* KO mice underwent water restriction and habituation before behavioral training (Figure 1A).

The odor-reward association task comprised four sequential stages (Figure 1A): Pavlovian acquisition (Pavl-Acq) and stabilization (Pavl-Stab) stages, followed by instrumental acquisition (Inst-Acq) and stabilization (Inst-Stab) stages. In each trial, a 1 s odor cue (0–1 s) was followed by a 2 s delay (1–3 s) and a 2 s response (3–5 s) window (Figure 1B). A trial was scored as a hit if the mouse licked within the response window (3–5 s), and as a miss otherwise (Figure 1B). In the Pavl stages, sucrose reward was delivered automatically at 3–3.5 s independent of behavior, whereas in the Inst stages, reward delivery was contingent on a licking response within the response window. Mice were required to reach a behavioral criterion (≥80% hit rate in two consecutive blocks) in each acquisition stage before advancing to the corresponding stabilization stage.

**Figure 2:**
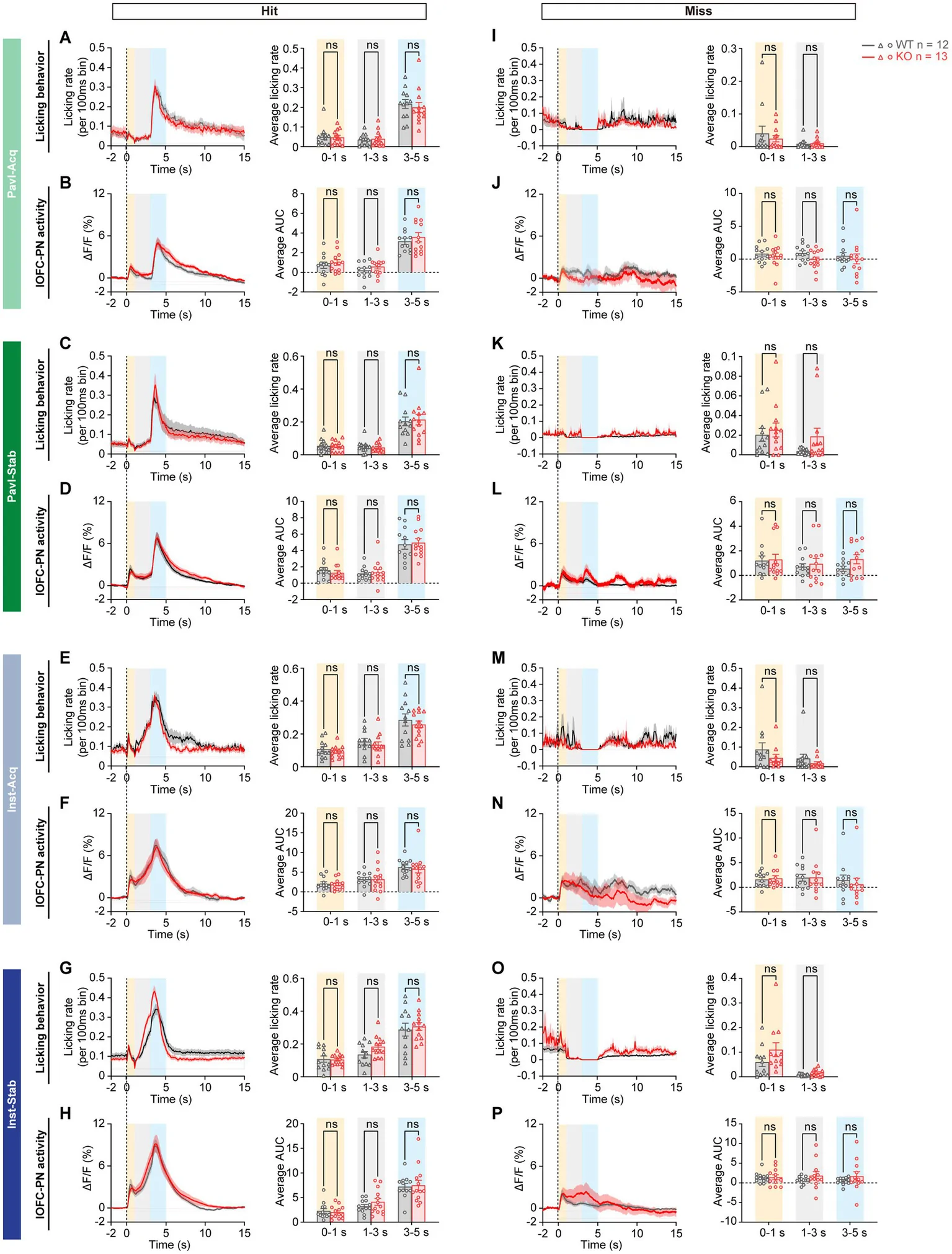
Licking behavior and lOFC-PN activity are comparable between WT and *Sapap3* KO mice across the four stages. **(A–H)** Licking behavior **(A,C,E,G)** and lOFC-PN calcium signals **(B,D,F,H)** during hit trials, shown for the Pavl-Acq **(A,B)**, Pavl-Stab **(C,D)**, Inst-Acq **(E,F)**, and Inst-Stab **(G,H)** stages. **(I–P)** Licking behavior **(I,K,M,O)** and lOFC-PN calcium signals **(J,L,N,P)** during miss trials, shown for the Pavl-Acq **(I,J)**, Pavl-Stab **(K,L)**, Inst-Acq **(M,N)**, and Inst-Stab **(O,P)** stages. Within each panel: Left, average lick rate or ΔF/F traces, with WT (*n* = 12) and *Sapap3* KO (*n* = 13) mice; right, average lick rate or average area under the curve (AUC) of ΔF/F within the odor (0–1 s, yellow), delay (1–3 s, gray), and response window (3–5 s, blue) epochs. Each symbol represents one mouse. Miss trials contained no licking during the response window by definition, and the 3–5 s epoch is therefore omitted from the licking quantification **(I,K,M,O)**. Shaded areas around traces represent SEM. Two-way ANOVA with Bonferroni’s multiple comparisons test. Data are mean ± SEM.

The number of blocks required to reach criterion did not differ between WT and KO mice in either the Pavl-Acq or Inst-Acq stage (Figure 1C), indicating comparable task acquisition across genotypes. We then examined licking behavior and lOFC-PN activity cross all four stages between WT and *Sapap3* KO mice (representative single-mouse traces shown in Figures 1D,E). Licking rate traces and lOFC-PN calcium signals were similar between WT and KO mice for both hit and miss trials (Figure 2). Quantification of average licking rate and area under the curve (AUC) of lOFC-PN signals within each task epoch (odor: 0–1 s; delay: 1–3 s; response window: 3–5 s) revealed no significant differences between genotypes in any stage or trial type (Figure 2). These results demonstrate that licking behavior and lOFC-PN activity were comparable between WT and *Sapap3* KO mice, supporting the pooling of WT and KO animals for all subsequent analyses.

### Result 2. Licking behavior and lOFC-PN activity differ between reward contingencies during hit trials

Having confirmed that WT and *Sapap3* KO mice were comparable, we pooled all 25 mice and compared licking behavior and lOFC-PN activity across the four training stages, separately for hit and miss trials.

**Figure 3:**
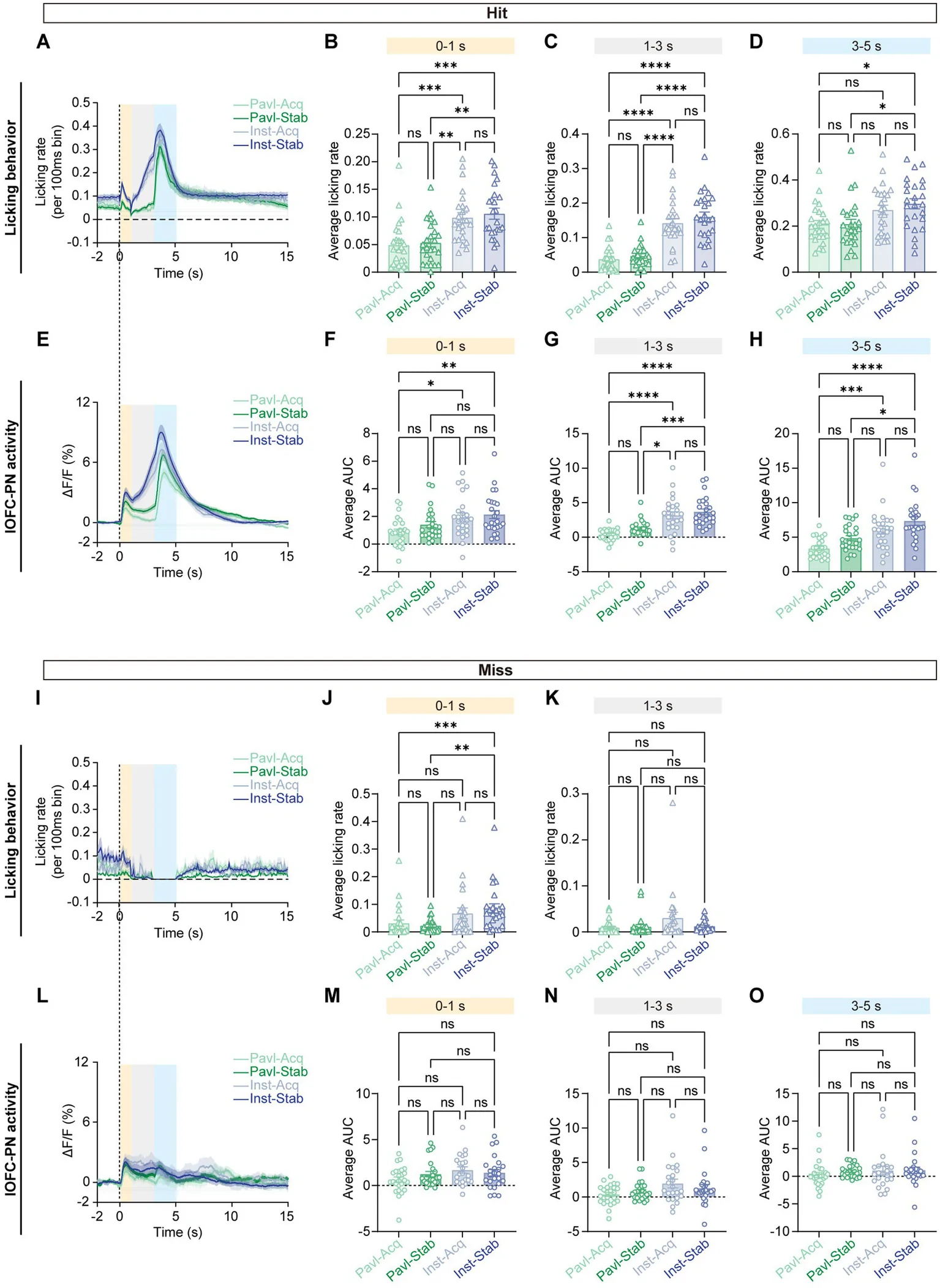
Licking behavior and lOFC-PN activity across the four stages in pooled mice **(A–H)** Hit trials. **(A)** Average licking rate traces, shown for Pavl-Acq (light green), Pavl-Stab (dark green), Inst-Acq (light blue), and Inst-Stab (dark blue) stages. Shaded areas around traces represent SEM. **(B–D)** Average licking rate quantified within the odor (**B**, 0–1 s), delay (**C**, 1–3 s), and response window (**D**, 3–5 s) epochs across the four stages. Each symbol represents one mouse. **(E)** Average lOFC-PN calcium signal traces across the four stages. **(F–H)** Average AUC of lOFC-PN activity quantified within the odor (**F**, 0–1 s), delay (**G**, 1–3 s), and response window (**H**, 3–5 s) epochs. **(I–O)** Miss trials. **(I)** Average licking rate traces across the four stages. **(J,K)** Average licking rate quantified within the odor (**J**, 0–1 s) and delay (**K**, 1–3 s) epochs. **(L)** Average lOFC-PN calcium signal traces across the four stages. **(M–O)** Average AUC of lOFC-PN calcium signal quantified within the odor (**M**, 0–1 s), delay (**N**, 1–3 s), and response window (**O**, 3–5 s) epochs. *n* = 25 mice. *p < 0.05, **p < 0.01, ***p < 0.001, ****p < 0.0001; Kruskal-Wallis test. Data are mean ± SEM.

In hit trials, both licking rate and lOFC-PN activity traces were similar between the two Pavlovian stages (Pavl-Acq and Pavl-Stab) and between the two instrumental stages (Inst-Acq and Inst-Stab), but differed markedly between Pavlovian and instrumental stages, particularly during the delay period (Figures 3A,E). Quantification confirmed no significant differences in licking rate or lOFC-PN AUC between Pavl-Acq and Pavl-Stab or between Inst-Acq and Inst-Stab in any epoch (Figures 3B–D). In contrast, licking rates were significantly higher in the instrumental than the Pavlovian stages during both the odor (0–1 s) and delay (1–3 s) epochs (Figures 3B,C). lOFC-PN signal AUC followed a similar pattern: no significant differences within the same contingency, but significantly higher activity in instrumental than Pavlovian stages across multiple epochs (Figures 3F–H).

In miss trials, both licking and lOFC-PN signal traces were overlapping across all four stages (Figures 3I,L). Licking rates showed only a significant elevation in Inst-Stab during the odor epoch (0–1 s) compared with the Pavlovian stages, with no differences in other epochs (Figures 3J,K). Thus, despite a statistically significant difference in licking during the odor epoch, lOFC-PN activity remained unchanged across stages (Figures 3L–O).

Together, these results demonstrate that licking behavior and lOFC-PN activity were stable within each contingency across acquisition and stabilization stages. While differences in licking between contingencies were observed in both hit and miss trials, differences in lOFC-PN activity between contingencies emerged specifically during hit trials, suggesting that the contingency-related differences in lOFC-PN activity were associated with successful reward acquisition rather than licking *per se*.

### Result 3. Differences in lOFC-PN activity between Pavlovian and instrumental phases during hit trials but not miss trials

Given that licking behavior and lOFC-PN activity were stable across acquisition and stabilization within each contingency (Figure 3), we merged stages sharing the same reward contingency into a Pavlovian phase (Pavl-Acq & Pavl-Stab) and an instrumental phase (Inst-Acq & Inst-Stab) for subsequent analyses.

**Figure 4:**
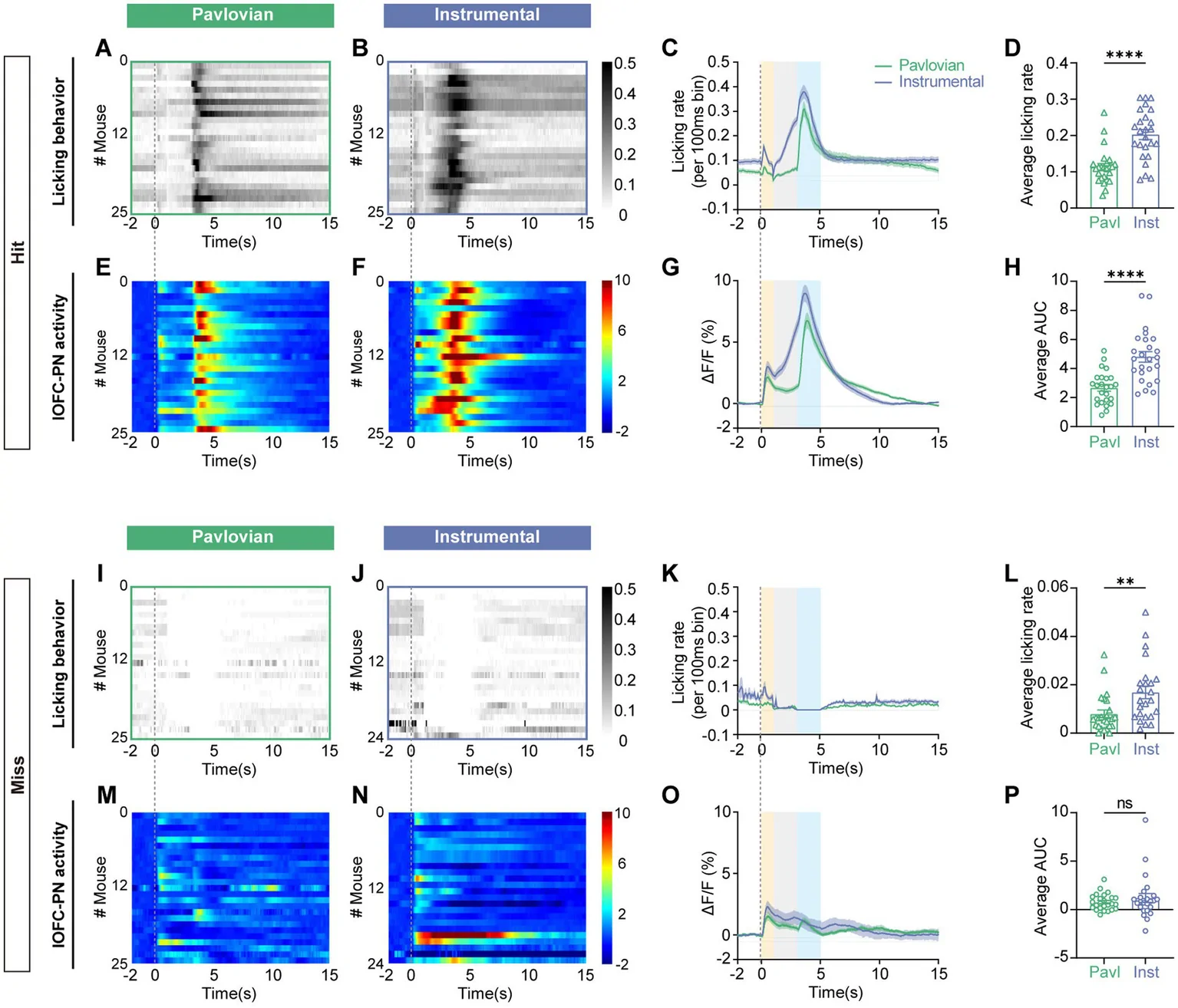
Comparison of licking behavior and lOFC-PN activity between Pavlovian and instrumental phases in pooled mice. **(A–H)** Hit trials. **(A,B)** Heatmaps of average licking rate (per 100 ms bin) per mouse, under Pavlovian **(A)** and instrumental **(B)** phases (*n* = 25 mice per phase). Each row represents one mouse. **(C)** Average licking rate traces across mice, under Pavlovian (green) and instrumental (blue) phases. Shaded regions along the time axis indicate task epochs (odor, 0–1 s, yellow; delay, 1–3 s, gray; response window, 3–5 s, blue). Shaded areas around traces represent SEM. **(D)** Average licking rate during the 0–5 s window, compared between Pavlovian and instrumental phases. Each symbol represents one mouse. **(E,F)** Heatmaps of lOFC-PN calcium signals per mouse, under Pavlovian **(E)** and instrumental **(F)** phases. **(G)** Average lOFC-PN calcium signal traces across mice. **(H)** AUC of lOFC-PN calcium signals during the 0–5 s window. **(I–P)** Miss trials, plotted as in **(A–H)**. Note that one mouse had no miss trials during the instrumental phase; *n* = 25 mice for Pavlovian and *n* = 24 mice for instrumental phases. *p < 0.05, **p < 0.01, ***p < 0.001, ****p < 0.0001; Mann–Whitney test. Data are mean ± SEM.

In hit trials (Figures 4A–H), both licking rate and lOFC-PN signal AUC over the 0–5 s window were significantly higher in the instrumental than the Pavlovian phase (Figures 4D,H). In miss trials (Figures 4I–P), although licking rate was also significantly higher in the instrumental than the Pavlovian phase (Figure 4L), lOFC-PN signal AUC showed no significant difference between phases (Figure 4P).

The phase-level analysis thus confirms and extends the stage-level findings: contingency-related differences in lOFC-PN activity were observed in hit trials but not miss trials, despite contingency-related differences in licking being present in both. This dissociation suggests that the contingency-related differences in lOFC-PN activity were associated with successful reward acquisition rather than with licking *per se*.

### Result 4. A cue-free lick test reveals that lOFC-PN activity is driven by reward-paired licking rather than licking *per se*

**Figure 5:**
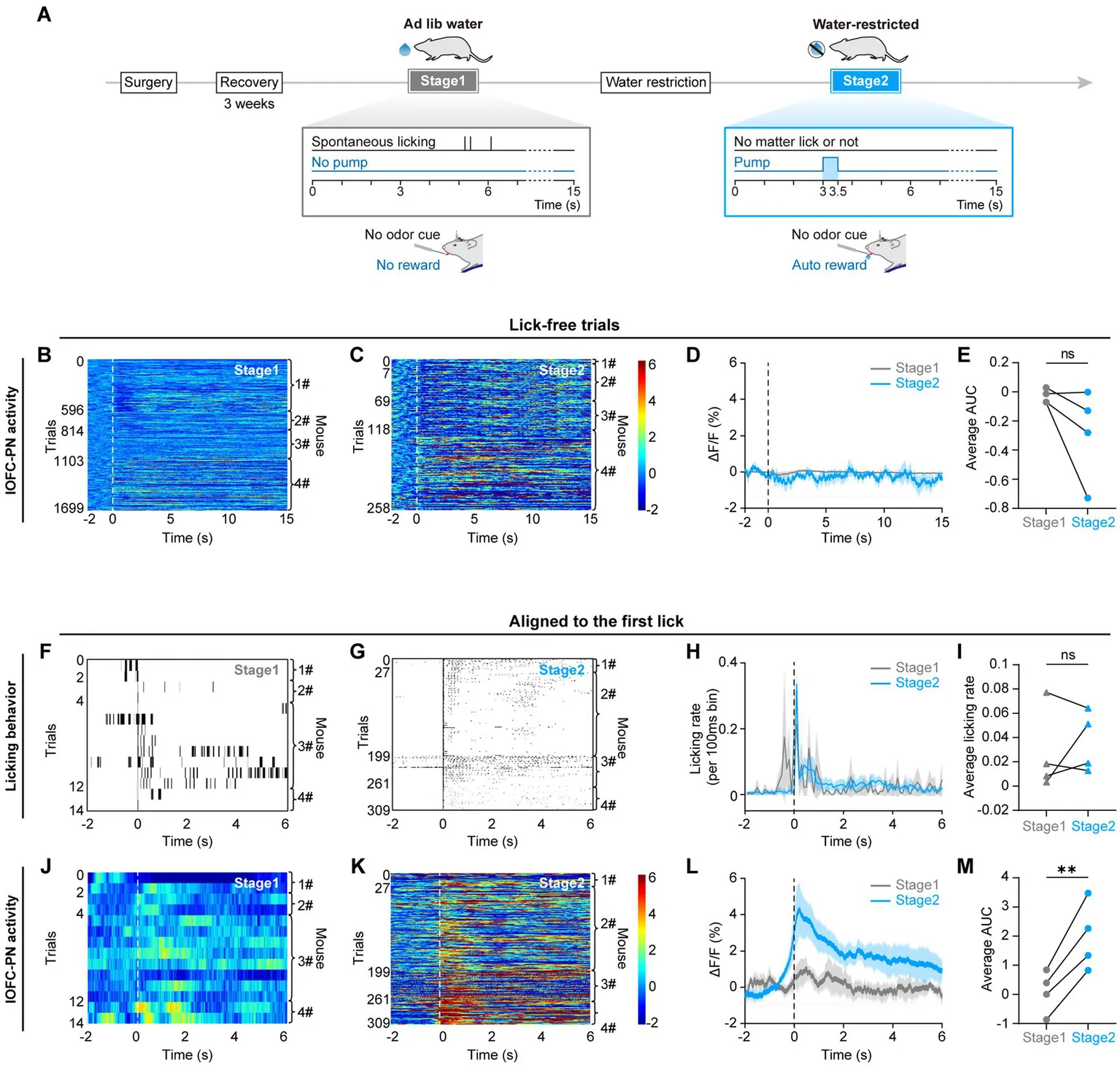
Cue-free lick test reveals that lOFC-PN activity is driven by reward-paired licking rather than licking *per se*. **(A)** Schematic of the two-stage cue-free lick test. **(B,C)** Heatmaps of lOFC-PN calcium signals (ΔF/F) during lick-free trials across the four mice in Stage 1 **(B)** and Stage 2 **(C)**. Trials are stacked by mouse (boundaries indicated on the right axis), and time is aligned to trial onset. **(D)** Mean lOFC-PN calcium signals (ΔF/F) during lick-free trials, averaged across the four mice in Stage 1 (gray) and Stage 2 (blue). Shaded areas indicate SEM. **(E)** Average AUC of calcium signals during 0–15 s of lick-free trials, *n* = 4 mice, paired *t* test, *p* = 0.17. **(F,G)** Lick raster plots of trials containing licking events, aligned to the first lick, in Stage 1 **(F)** and Stage 2 **(G)**. The relatively small number of Stage 1 trials reflects the low spontaneous licking rate under ad libitum water conditions. **(H)** Mean licking rate (per 100 ms bin) following the first lick, averaged across the four mice in Stage 1 (gray) and Stage 2 (blue). **(I)** Average licking rate during 0–6 s following the first lick, *n* = 4 mice, paired *t* test, *p* = 0.52. **(J,K)** Heatmaps of lOFC-PN calcium signals during trials containing licking events, aligned to the first lick, in Stage 1 **(J)** and Stage 2 **(K)**. **(L)** Mean lOFC-PN calcium signals following the first lick, averaged across the four mice in Stage 1 (gray) and Stage 2 (blue). **(M)** Average AUC of calcium signals during 0–6 s following the first lick, *n* = 4 mice, paired *t* test, *p* = 0.0063. *p* < 0.01.

Although the hit and miss dissociation suggested that licking *per se* could not fully explain the contingency-related differences in lOFC-PN activity (Figures 3,4), licking might still contribute to these differences within hit trials, where licking rates also differed between contingencies. To directly test whether licking *per se* is sufficient to drive lOFC-PN activity, we performed a cue-free lick test in a separate cohort of WT mice across two consecutive stages in which licking occurred either without or with reward: in Stage 1, licking occurred without reward (spontaneous licking under ad libitum water); in Stage 2, licking occurred with reward (cue-free automatic reward delivery under water restriction) (Figure 5A).

We first compared lOFC-PN activity during lick-free trials between the two stages. lOFC-PN signals remained near baseline in both stages, with no significant difference in AUC (Figures 5B–E), indicating that the difference in motivational state between stages did not alter lOFC-PN activity.

We next examined trials containing licking events, aligned to the first lick. Licking rates following the first lick were comparable between the two stages (Figures 5F–I). Despite this similarity in licking behavior, lOFC-PN activity diverged sharply between stages. In Stage 1, where licking was unpaired with reward, calcium signals remained near baseline following the first lick (Figures 5J,L). In Stage 2, where licking was followed by reward, robust calcium transients emerged (Figures 5K,L), with significantly higher AUC in Stage 2 (Figure 5M).

Together, these results demonstrate that licking *per se* is not sufficient to drive lOFC-PN activity. Instead, lOFC-PN activity is selectively engaged when licking is paired with reward, supporting the interpretation that the contingency-related differences in lOFC-PN activity observed in our main task (Figures 3, 4) reflect reward-related processing rather than licking motor activity.

### Result 5. Linear mixed-effects modeling identifies reward contingency as an independent contributor to lOFC-PN activity beyond licking

**Figure 6:**
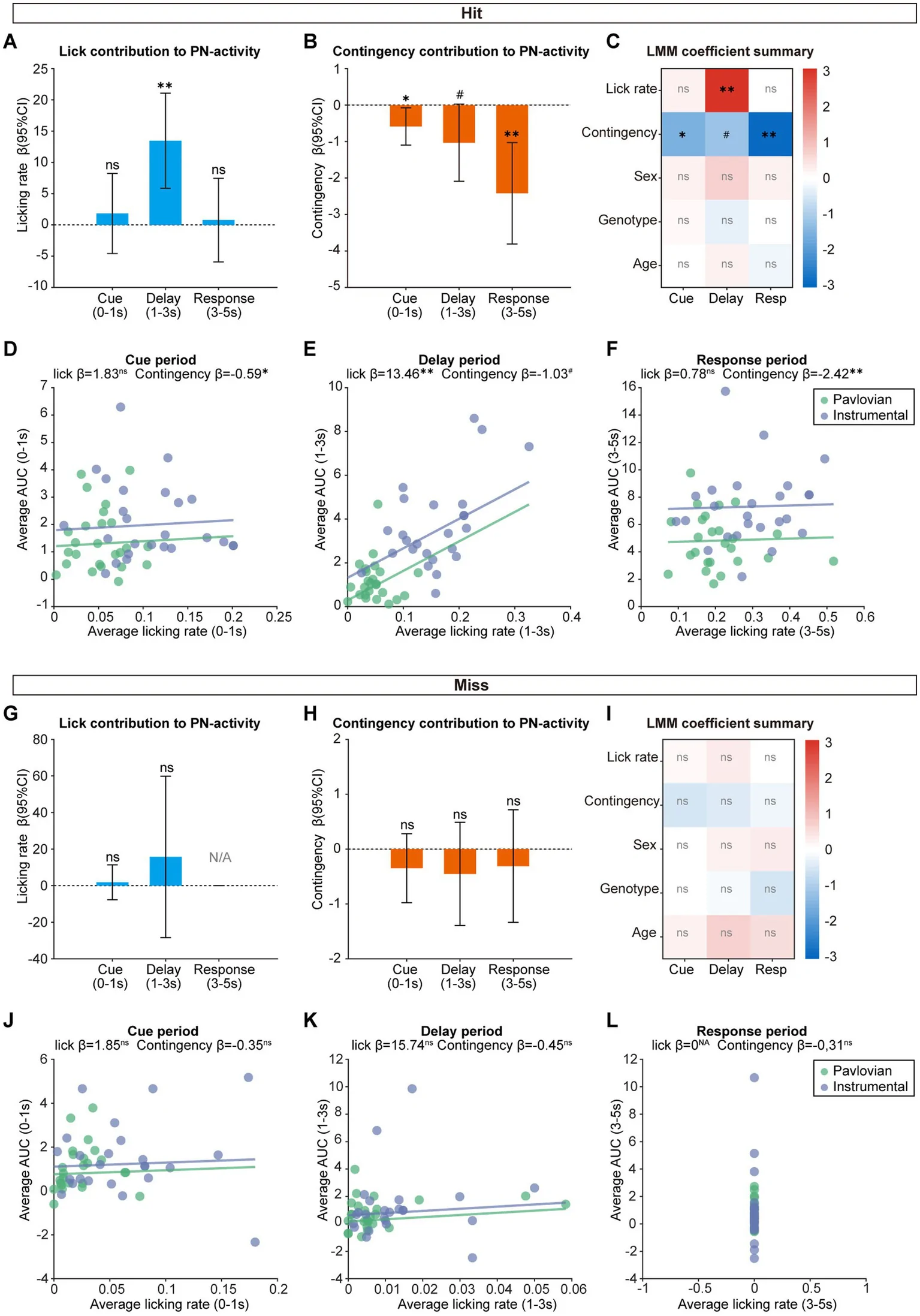
Linear mixed-effects modeling (LMM) dissociates reward contingency from licking as contributors to lOFC-PN activity. **(A–F)** LMM analysis of hit trials (*n* = 50 observations from 25 mice × 2 phases). Contingency was coded with instrumental as the reference category; negative *β* indicates higher *AUC* under instrumental than Pavlovian contingencies. **(A)** Regression coefficients (*β*) of lick rate as a predictor of lOFC-PN *AUC* during the cue (0–1 s), delay (1–3 s), and response (3–5 s) epochs. Error bars represent 95% confidence intervals. **(B)** *β* of reward contingency as a predictor of lOFC-PN *AUC* during the three epochs. **(C)** Heatmap summary of LMM coefficients for all five fixed-effect predictors (lick rate, contingency, sex, genotype, age) across the three epochs. Color represents signed -log₁₀(*p*), where the sign indicates the direction of *β* (red = positive, blue = negative). **(D–F)** Scatter plots of average lOFC-PN *AUC* vs. licking rate within each epoch (**D**, Cue; **E**, Delay; **F**, Response), for Pavlovian (green) and instrumental (blue) phases. Each point represents one mouse-phase observation. Lines indicate model-predicted relationships. **(G–L)** LMM analysis of miss trials (*n* = 49 observations; one mouse had no miss trials during the instrumental phase). By definition, miss trials in the response epoch contained no licking events; the lick rate predictor was therefore omitted from the response-epoch model. **(G)** *β* of lick rate as a predictor of lOFC-PN *AUC* during the cue and delay epochs (lick rate was not included in the response-epoch model, indicated as N/A). **(H)** *β* of reward contingency as a predictor of lOFC-PN *AUC* across the three epochs. **(I)** Heatmap summary of LMM coefficients for miss trials, as in **(C)**. **(J–L)** Scatter plots as in **(D–F)**, for miss trials (**J**, Cue; **K**, Delay; **L**, Response; all points in **L** have lick rate = 0 by definition). #*p* < 0.1 (trend-level), *p < 0.05, **p < 0.01, ***p < 0.001.

To further dissociate the contribution of licking from that of reward contingency in driving lOFC-PN activity, while controlling for sex, genotype, age, and between-subject variability, we performed linear mixed-effects modeling (LMM) on the calcium AUC of each task epoch (odor: 0–1 s; delay: 1–3 s; response window: 3–5 s; Figure 6).

In hit trials, lick rate significantly contributed to lOFC-PN AUC only during the delay epoch (*β* = 13.46, *p* = 0.0012), but not during the cue (*β* = 1.83, *p* = 0.74) or response (*β* = 0.78, *p* = 0.82) epochs (Figures 6A,D–F). In contrast, reward contingency significantly contributed to lOFC-PN AUC during the cue and response epochs, with a trend-level contribution during the delay epoch (cue: *β* = −0.59, *p* = 0.028; delay: *β* = −1.03, *p* = 0.057; response: *β* = −2.42, *p* = 0.0014; Figures 6B,D–F). Notably, in both the cue and response epochs, contingency was a significant predictor of lOFC-PN activity while lick rate was not, demonstrating that the contingency-related differences in lOFC-PN activity cannot be explained by licking behavior alone. Sex, genotype, and age showed no significant contribution in any epoch (Figure 6C).

In miss trials, no variable significantly contributed to lOFC-PN AUC in any epoch (Figures 6G–L), consistent with the absence of contingency-related modulation of lOFC-PN activity during unsuccessful trials observed in Figures 3, 4.

Together, these LMM results demonstrate that reward contingency contributes to lOFC-PN activity independently of licking, sex, genotype, and age, confirming that the contingency-specific differences in lOFC-PN activity reflect reward-related processing rather than licking motor activity *per se*.

### Result 6. lOFC-PN activity timing becomes coupled to anticipatory licking only under instrumental contingency

**Figure 7:**
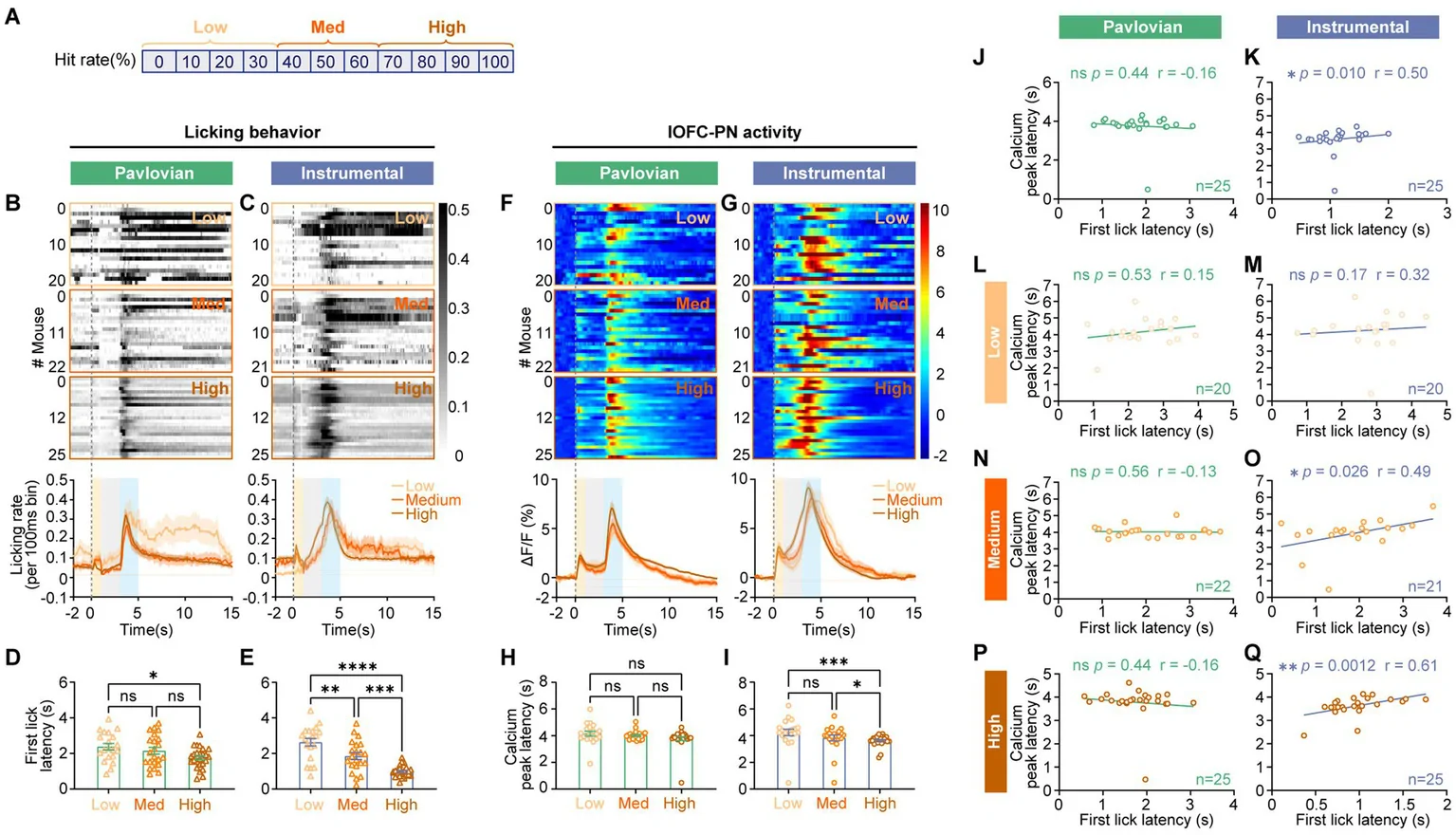
Calcium peak latency becomes coupled to first lick latency only under instrumental contingencies as learning progresses. **(A)** Schematic of behavioral block stratification by hit rate into three performance levels: Low (≤30%), Medium (40–60%), and High (≥70%). **(B,C)** Licking behavior across performance levels under Pavlovian **(B)** and instrumental **(C)** contingencies. Top: Heatmaps of average licking rate (per 100 ms bin) per mouse, with each row representing one mouse at the corresponding performance level. The number of mice contributing to each level varies because not all mice had blocks in every performance range. Bottom: Average licking rate traces across mice for Low, Medium, and High performance. Shaded regions along the time axis indicate task epochs (cue, 0–1 s, yellow; delay, 1–3 s, gray; response, 3–5 s, blue). Shaded areas around traces represent SEM. **(D,E)** First lick latency at each performance level, under Pavlovian (**D**, *p* = 0.027) and instrumental (**E**, *p* < 0.0001) contingencies. Each symbol represents one mouse at the corresponding performance level. One-way ANOVA. **(F,G)** Heatmaps (top) and traces (bottom) of lOFC-PN calcium signals across performance levels under Pavlovian **(F)** and instrumental **(G)** contingencies. **(H,I)** Calcium peak latency at each performance level, under Pavlovian (**H**, *p* = 0.080) and instrumental (**I**, *p* = 0.0002) contingencies. Kruskal-Wallis test. **(J,K)** Correlation between calcium peak latency and first lick latency, pooled across all performance levels (*n* = 25 mice). Pavlovian (**J**, *p* = 0.44, *r* = −0.16) and instrumental (**K**, *p* = 0.010, *r* = 0.50). Spearman correlation. **(L–Q)** Correlation between calcium peak latency and first lick latency, separated by performance level: Pavlovian (left) and instrumental (right). Low (**L**: *p* = 0.54, *r* = 0.15; **M**: *p* = 0.17, *r* = 0.32). Medium (**N**: *p* = 0.56, *r* = −0.13; **O**: *p* = 0.026, *r* = 0.49). High (**P**: *p* = 0.44, *r* = −0.16; **Q**: *p* = 0.0012, *r* = 0.61). Spearman correlation. *p < 0.05, **p < 0.01, ***p < 0.001, ****p < 0.0001.

While the LMM analysis identified the contributions of contingency and licking to overall lOFC-PN activity within each task epoch (Figure 6), it did not address how the timing of lOFC-PN activity relates to behavioral timing across learning. To examine this, we stratified behavioral blocks by hit rate (Low: ≤30%, Medium: 40–60%, High: ≥70%) (Figure 7A), and analyzed first lick latency and calcium peak latency from hit trials within each performance level in each phase.

First lick latency decreased progressively with performance in both Pavlovian and instrumental phases (Figures 7B–E), indicating that mice initiated anticipatory licking earlier as learning advanced. Calcium peak latency, however, showed contingency-dependent patterns within each phase (Figures 7F–I). In the Pavlovian phase, calcium peak latency remained stable around 4 s across all performance levels, coinciding with reward delivery (Figures 7F,H). In the instrumental phase, calcium peak latency progressively advanced with performance, reaching significantly earlier values at High than at Low or Medium performance (Figures 7G,I).

To directly test whether the timing of lOFC-PN activity was coupled to that of anticipatory licking, we correlated calcium peak latency with first lick latency across mice. In the Pavlovian phase, no significant correlation was detected, either overall (Figure 7J) or at any performance level (Figures 7L,N,P), indicating that lOFC-PN activity timing remained independent of licking timing throughout learning. In the instrumental phase, by contrast, the correlation emerged and strengthened progressively with learning: no correlation was detected at Low performance (Figure 7M), a significant correlation emerged at Medium performance (Figure 7O), and the correlation further strengthened at High performance (Figure 7Q). Pooling across performance levels confirmed an overall significant correlation in the instrumental phase (Figure 7K).

Together, these results reveal a contingency-dependent shift in the temporal coupling of lOFC-PN activity. Under Pavlovian contingencies, lOFC-PN activity timing remained aligned with reward delivery and independent of licking initiation. Under instrumental contingencies, lOFC-PN activity became progressively coupled to anticipatory licking, reflecting a shift from reward-aligned to action-aligned lOFC-PN activity.

### Result 7. Performance-related lOFC-PN activity is concentrated in distinct task epochs under Pavlovian and instrumental reward contingencies

**Figure 8:**
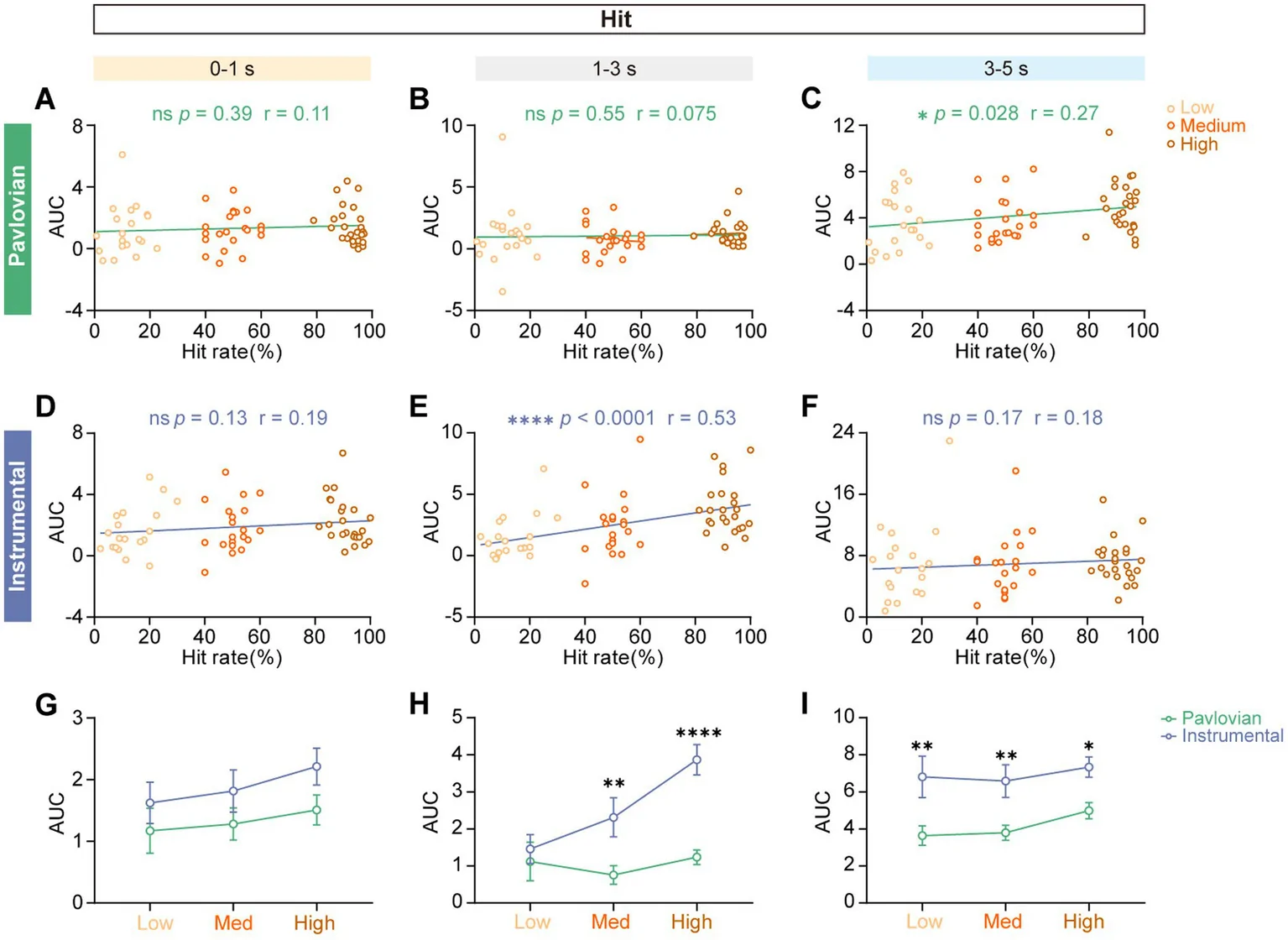
Performance-related lOFC-PN activity is concentrated in distinct temporal epochs under Pavlovian and instrumental contingencies. **(A–F)** Correlations between hit rate and lOFC-PN AUC within each task epoch (cue: 0–1 s; delay: 1–3 s; response: 3–5 s), separately for Pavlovian **(A–C)** and instrumental **(D–F)** phases. Each point represents one mouse at specific performance level (Low, Medium, or High). Lines indicate linear regression fits. *r* and *p* values are shown on each panel; Spearman correlation. **(G–I)** Comparison of average lOFC-PN AUC across performance levels between Pavlovian and instrumental phases, within the cue **(G)**, delay **(H)**, and response **(I)** task epochs. Mixed-effects analysis. *p < 0.05, **p < 0.01, ***p < 0.001, ****p < 0.0001. Data are mean ± SEM.

Having shown that the timing of lOFC-PN activity becomes contingency-dependent (Figure 7), we next asked in which task epoch lOFC-PN activity tracks behavioral performance under each contingency. To address this, we correlated hit rate with lOFC-PN activity in each task epoch (cue: 0–1 s; delay: 1–3 s; response: 3–5 s) separately for each phase (Figures 8A–F).

In the Pavlovian phase, lOFC-PN AUC correlated significantly with hit rate only during the response epoch (Figure 8C), with no significant correlation during the cue (Figure 8A) or delay (Figure 8B) epochs. In the instrumental phase, by contrast, lOFC-PN AUC correlated significantly with hit rate only during the delay epoch (Figure 8E), with no significant correlation during the cue (Figure 8D) or response (Figure 8F) epochs. Thus, the performance-related lOFC-PN activity was specific to the reward delivery period under Pavlovian contingencies, but shifted to the pre-reward delay period under instrumental contingencies.

Between-phase comparison further revealed that lOFC-PN AUC was significantly higher in the instrumental than the Pavlovian phase at Medium and High performance during the delay epoch (Figure 8H), and at all three performance levels during the response epoch (Figure 8I), with no significant differences during the cue epoch (Figure 8G).

Together, these results reveal contingency-specific task epochs in which performance-related lOFC-PN activity is concentrated. Under Pavlovian contingencies, this activity tracked performance during the reward delivery epoch. Under instrumental contingencies, it tracked performance during the anticipatory delay period. Combined with the temporal coupling shown in Figure 7, these findings collectively indicate a shift from reward-aligned to action-aligned lOFC-PN activity.

## Discussion

**Figure 9:**
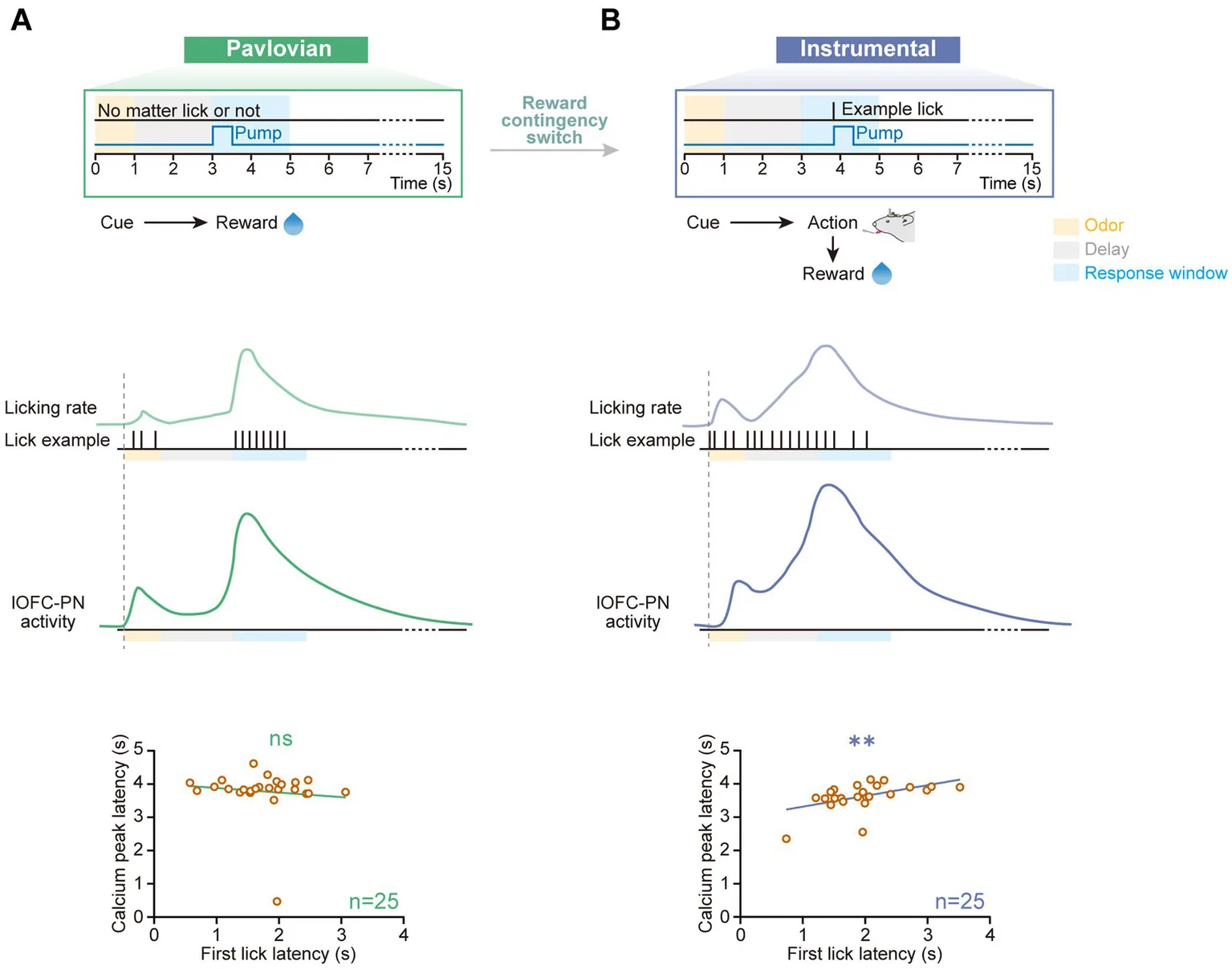
Working model of contingency-dependent reorganization of lOFC-PN activity from reward-aligned to action-aligned. **(A, B)** Schematic illustrating the proposed working model under Pavlovian **(A)** and instrumental **(B)** reward contingencies. Top: Task structure. Middle: Representative licking rate (top trace) and lOFC-PN calcium signal (bottom trace), with lick raster examples in between. Bottom: Scatter plots showing the relationship between first lick latency (x-axis) and calcium peak latency (y-axis) at high performance level **(I)** (*n* = 25 mice per phase). Under Pavlovian **(A)** and instrumental **(B)** contingencies. Yellow shading, odor delivery (0–1 s); gray shading, delay period (1–3 s); blue shading, response window (3–5 s). Ns, not significant; **p < 0.01.

Our results support a working model in which reward contingency is associated with distinct temporal patterns of both licking behavior and lOFC-PN activity during associative learning (Figure 9). Under Pavlovian contingencies, where reward delivery was independent of behavior, licking was concentrated around the time of reward delivery within the response window, and performance-related lOFC-PN activity was similarly concentrated around the response window; no significant temporal coupling between neural activity and anticipatory licking was detected (Figure 9A). In contrast, under instrumental contingencies, where reward was contingent on the animal’s action, anticipatory licking emerged progressively during the pre-reward delay period, and lOFC-PN activity also extended into the delay period in parallel with the licking pattern; its timing became progressively coupled to anticipatory licking with learning (Figure 9B). Together, these results identify two distinct temporal patterns of both licking behavior and lOFC-PN activity: reward-aligned mode under Pavlovian learning and action-aligned mode under instrumental learning.

A potential concern arising from our findings is whether the contingency-related differences in lOFC-PN activity simply reflect the differences in licking behavior between the two contingencies, rather than reward-related processing. This concern is reinforced by recent work showing that movement-related signals can pervade multiple brain regions (Musall et al., 2019; Stringer et al., 2019). Two complementary lines of evidence argue against a pure licking account. Experimentally, the cue-free lick test (Figure 5) showed that licking in the absence of motivational drive and reward did not elicit detectable lOFC-PN activity, whereas licking in the presence of motivational drive and reward was accompanied by robust lOFC-PN responses, despite comparable licking dynamics across the two conditions. Statistically, the linear mixed-effects model (Figure 6) revealed that reward contingency contributed to lOFC-PN activity independently of licking during the cue and response epochs, where contingency was a significant predictor while lick rate was not. In the delay epoch, by contrast, lick rate was the primary contributor; this pattern, however, does not undermine the broader interpretation, as the close coupling between delay-epoch activity and anticipatory licking is itself a defining feature of action-aligned activity under instrumental contingencies. Together, these experimental and statistical results indicate that licking *per se* was not sufficient to drive lOFC-PN activity. Rather, lOFC-PN activity emerged when licking occurred in the context of reward-related processing, encompassing motivational drive, reward expectation, and anticipatory action.

The contingency-dependent shift in the temporal alignment of lOFC-PN activity raises the question of how this pattern relates to existing accounts of lOFC function. Prior work has characterized the lOFC as a central node in reward-based learning by emphasizing its role in encoding expected outcomes, reward value, and task states (Schoenbaum et al., 2009; Wallis, 2007; Ogg et al., 2025; Maier, 2015; Walton et al., 2010). In these accounts, however, contingency structure typically co-varied with other task features, leaving its specific contribution to the temporal organization of lOFC activity unresolved. Our task design isolated this contribution and revealed that lOFC-PN activity can dynamically reorganize its temporal alignment with behavior depending on whether reward acquisition is passive or action-dependent.

Our cohort included both WT and *Sapap3* KO mice, allowing us to assess whether this strain shows abnormalities in our simple odor-reward task. Licking behavior and lOFC-PN activity were comparable between genotypes across all stages and trial types (Figures 1, 2), consistent with prior reports of intact odor discrimination in *Sapap3* KO mice (Yang et al., 2021). Previous work has reported impairments in *Sapap3* KO mice during reversal learning (Yang et al., 2021; Manning et al., 2023; Benzina et al., 2021; van den Boom et al., 2019; Manning et al., 2019), a paradigm requiring reversal of an established stimulus-outcome association to assess cognitive flexibility. Our task differs from reversal learning in this regard. Although it involves a change from passive to action-dependent reward acquisition, the stimulus-outcome association itself remains intact, since the cue continues to predict reward across both contingencies. Our findings therefore indicate that this paradigm does not engage the cognitive processes selectively disrupted in *Sapap3* KO mice, complementing prior literature focused on reversal learning.

One limitation of our study is that all animals were trained sequentially on the Pavlovian and instrumental phases, leaving contingency partially confounded with training history. Nevertheless, two features of our data argue against a pure training-history account. The contingency-related differences in lOFC-PN activity were detected during hit trials but not during miss trials (Figures 3, 4), whereas a generalized training-history effect would be expected to affect both trial types. In addition, the cue-free lick test (Figure 5) was conducted in a separate cohort and does not depend on the Pavlovian-to-instrumental order. Future studies using counterbalanced training designs or a naïve instrumental cohort will nonetheless be needed to fully dissociate contingency from training history.

A further consideration relates to the simplicity of our paradigm, which constrained how learning could be analyzed. Mice rapidly reached the behavioral criterion (≥80% hit rate in two consecutive blocks), precluding continuous temporal analysis of how behavior and lOFC-PN activity evolved across learning. We therefore used block-level hit rate as a performance metric and stratified blocks into Low/Medium/High bins, a tripartite scheme designed to capture how lOFC-PN activity varies across distinct levels of behavioral performance while maintaining sufficient blocks per bin. Future work incorporating greater task complexity, such as probabilistic reinforcement or graded reward magnitudes, would slow acquisition and enable continuous, trial-by-trial modeling of learning trajectories.

## Conclusion

In summary, by isolating contingency structure from stimulus identity and reward value, our task design reveals that reward contingency is associated with parallel temporal reorganization of both anticipatory licking behavior and lOFC-PN activity, shifting from reward-aligned under Pavlovian learning to action-aligned under instrumental learning. As learning advanced, lOFC-PN activity timing became progressively coupled to anticipatory licking under instrumental but not Pavlovian contingencies, and this contingency-dependent reorganization could not be accounted for by licking *per se*, indicating that lOFC-PN activity reflects reward-related processing rather than motor output. Together, these findings identify reward contingency as a key determinant of how lOFC pyramidal neurons temporally align their activity with behavior during associative learning, with implications for understanding how the OFC supports flexible coordination between learning and goal-directed action.

## Materials and methods

### Animals

Mice were housed under SPF conditions at 24 ± 2 °C with 45–65% humidity. Age- and sex-matched mice aged 3–6 months were used for all experiments. Prior to behavioral testing, mice were maintained on a 12 h reversed light/dark cycle for at least 4 weeks. *Sapap3* knockout (KO) mice were obtained from the laboratory of Guoping Feng (MIT). All behavioral testing was conducted during the mice’s active (dark) phase.

### Stereotaxic surgery

#### Virus injection

Surgical procedures followed established stereotaxic methods described in our previous work (Yang et al., 2021). In brief, mice were anesthetized with tribromoethanol (0.25–0.3 mg/g, i.p.) and head-fixed in a stereotaxic frame (Siskiyou). Body temperature was maintained at 37 °C throughout the procedure using a heating pad (ThinkerTech) and ophthalmic ointment was applied to protect the corneas. A small craniotomy was made over the lOFC using a dental drill (Foredom) at the following coordinates relative to bregma: AP + 2.65 mm, ML ± 1.45 mm, DV -1.50 mm. AAV2/9-CaMKIIα-GCaMP6m (2–5 × 10^12^ viral genomes/ml; Shanghai Taitool Bioscience) was injected at a volume of 250 nL and a rate of 2 nL/s using a Nanoliter 2010 injector (WPI). The injection needle was left in place for 10 min after delivery.

### Optic fiber implantation

An optical fiber (200 μm OD, NA 0.37) was lowered to 100 μm above the injection site and secured to the skull with dental adhesive (C&B Metabond, Parkell). A custom-made stainless steel head plate was additionally affixed to the skull with Metabond to allow subsequent head fixation during behavioral testing. Carprofen (5 mg/kg, s.c.) was administered immediately after surgery for postoperative analgesia. Mice were singly housed for a minimum of 3 weeks to allow for full surgical recovery and viral expression before behavioral training commenced.

### Behavioral test

The experimental timeline is illustrated in Figure 1A. Following surgical recovery, mice were habituated to head fixation on a running wheel for 1–2 h per day over 3 consecutive days. Water restriction began 24–36 h before behavioral training and was maintained throughout the experiment to keep body weight at 85–90% of pre-restriction levels. Odors (clove) were delivered through computer-controlled valves. The licking spout was placed 1 mm below the lower lip and 3 mm caudal to the nasal tip to ensure reliable spout contact while minimizing non-task-related licking.

The odor-reward association task consisted of four sequential stages (Figure 1A). Each block comprised 10 trials, and the inter-trial interval was 15 s. Each trial began with a 1 s odor cue (0–1 s), followed by a 2 s delay period (1–3 s) and a 2 s response window (3–5 s). A trial was scored as a hit if the mouse licked the spout within the response window (3–5 s), or as a miss otherwise (Figure 1B). The hit rate per block was calculated as the percentage of hit trials out of 10 trials. Criterion was defined as a hit rate of ≥80% in two consecutive blocks.

In the Pavlovian acquisition (Pavl-Acq) stage, sucrose reward (5 μL) was delivered automatically at 3–3.5 s after trial onset, independent of the animal’s behavior. Mice advanced to the Pavlovian stabilization (Pavl-Stab) stage upon reaching criterion, during which training continued under the same contingency. In the instrumental acquisition (Inst-Acq) stage, reward delivery was contingent on the mouse licking the spout within the response window; each trial allowed only one reward delivery. Mice then advanced to the instrumental stabilization (Inst-Stab) stage upon reaching criterion.

First lick latency was defined as the time from trial onset to the first lick within a given trial. Calcium peak latency was defined as the time from trial onset to the peak of the calcium signal within a trial. Behavioral training was controlled using LabVIEW software and data were analyzed using custom MATLAB scripts.^1^.

### Cue-free lick test

A separate cohort of four adult wild-type C57BL/6 J mice underwent the same surgical procedures as the main cohort, receiving AAV-CaMKIIα-GCaMP6m injection into the lOFC and optical fiber implantation over the injection site. After 3 weeks of viral expression and recovery, mice underwent a two-stage cue-free lick test (Figure 5A). No odor cue was delivered in either stage. Each trial lasted 15 s.

In Stage 1, mice had ad libitum access to water in the home cage and were head-fixed on a running wheel for one 1-h session per day across three consecutive days. No sucrose reward was delivered. In Stage 2, mice were water-restricted for 36 h and then underwent a single 1-h session of head-fixed recording. At 3–3.5 s of each trial, 3 μL of 10% sucrose solution was automatically delivered through the lick spout, independent of the animal’s behavior.

Lick-free trials were defined as trials with no licking events across the 0–15 s window. Trials containing licking events were aligned to the first lick. In Stage 1, trials with the first lick occurring within 0–9 s were included; in Stage 2, trials with the first lick occurring within 3–5 s were included. The −2 to 0 s window was taken as baseline and the 0 to 6 s window as the response signal.

### Fiber photometry

Calcium-dependent fluorescence signals from lOFC pyramidal neurons were recorded using a fiber photometry system (ThinkerTech Nanjing Bioscience) as previously described (Yang et al., 2021; Li et al., 2019). A 470 nm laser (20–30 μW at the fiber tip) was used to excite GCaMP6m fluorescence. Signals were acquired at 100 Hz. Fluorescence was normalized to baseline using the formula:

$$\mathrm{\Delta F}/F\;(\%)=(F-F_{0})/(F_{0}-F_{\text{offset}})\times 100\%$$

Where F is the fluorescence during the behavioral event, F_0_ is the mean fluorescence during the 2 s preceding the event, and F_offset_ is the baseline offset recorded when the optical fiber was disconnected.

### Histology

Mice were anesthetized with a lethal dose of tribromoethanol (0.4 mg/kg, i.p.) and perfused transcardially with ice-cold PBS followed by 4% paraformaldehyde (PFA) in PBS. Brains were extracted and post-fixed in 4% PFA at 4 °C overnight, then transferred to 30% sucrose in PBS for cryoprotection until fully saturated. Coronal sections (30 μm thickness) through the virus injection site were cut on a cryostat (CM3050 S, Leica). For nuclear counterstaining, sections were incubated with Hoechst dye (1:5000, C1011, Beyotime) for 10 min at room temperature and imaged on a fluorescence microscope (ECLIPSE Ni-E, Nikon) using a 10 × objective (N. A. 0.3, W. D. 16.00 mm).

### Linear mixed-effects modeling

Linear mixed-effects modeling (LMM) was performed on the calcium AUC of each task epoch (cue: 0–1 s; delay: 1–3 s; response: 3–5 s), separately for hit and miss trials.

For each mouse, AUC and licking rate within each epoch were averaged across all trials of a given trial type (hit or miss) within each phase (Pavlovian or instrumental), yielding one observation per mouse per phase. AUC was computed from ΔF/F signals as described above.

For each epoch and trial type, the AUC was modeled as a function of lick rate in that epoch, reward contingency (Pavlovian vs. instrumental), sex, genotype (WT vs. KO), and age (categorized as young vs. old) as fixed effects, with mouse identity as a random intercept: AUC_epoch ~ lick_epoch + Contingency + Sex + Genotype + Age + (1 | Mouse).

Six independent models were fitted in total (3 epochs × 2 trial types). For miss trials, licking events were absent during the response epoch by definition, and the lick rate predictor was omitted from the response-epoch miss-trial model. All categorical predictors were entered as binary factors, with the alphabetically first level treated as the reference (Sex: F as reference; Genotype: KO as reference; Age: old as reference; Contingency: instrumental as reference). Models were fitted in MATLAB using the fitlme function with default restricted maximum likelihood (REML) estimation. For each predictor, the regression coefficient (*β*), 95% confidence interval, and *p*-value were extracted; significance levels were defined as *p* < 0.001 (***), *p* < 0.01 (**), *p* < 0.05 (*), and 0.05 ≤ *p* < 0.1 (#, trend-level).

### Quantification and statistical analysis

Behavioral and fiber photometry data were analyzed using custom MATLAB scripts (Z. Y.). Statistical analyses were performed in GraphPad Prism 9. Normality was assessed using the Shapiro–Wilk test. For normally distributed data, two-tailed unpaired t-tests, repeated-measures one-way ANOVA with Dunnett’s *post hoc* test, and Pearson correlation were used. For non-normally distributed data, Mann–Whitney tests, Wilcoxon matched-pairs signed rank test, Friedman tests, and Spearman correlation were used. Because missing values were present in certain accuracy levels or trial types in two-factor comparisons, mixed-effects analysis with Bonferroni’s multiple comparisons test was applied. Values are expressed as mean ± SEM. Statistical significance was set at *p* < 0.05. Detailed statistics are provided in the Statistical Table.

## Data Availability

The original contributions presented in the study are included in the article/Supplementary material, further inquiries can be directed to the corresponding author/s.

## Footnotes

1 Available at https://github.com/zichengyang/imaging_behavior.
