# Correction: Reward contingency reorganizes lateral orbitofrontal cortex pyramidal neuron activity from reward-aligned to action-aligned during associative learning

**DOI:** 10.3389/fncel.2026.1987114

**Published:** 2026-09-11

**Authors:** Zicheng Yang, Xiaolan Chen, Xiaohong Sun, Huimeng Lei

**Affiliations:** School of Basic Medical Sciences, Beijing Key Laboratory of Neural Regeneration and Repair, Key Laboratory for Neurodegenerative Diseases of the Ministry of Education, Laboratory for Clinical Medicine, Capital Medical University, Beijing, China

**Keywords:** anticipatory licking, fiber photometry, instrumental conditioning, lateral orbitofrontal cortex, Pavlovian conditioning, pyramidal neuron, reward contingency

The funder [Chinese Institutes for Medical Research, Beijing, CX24PY03 to HL] was erroneously omitted.

The original version of this article has been updated.

